# Epinephrine nasal spray for the treatment of anaphylaxis: perspectives in pediatrics

**DOI:** 10.1097/ACI.0000000000001109

**Published:** 2025-09-16

**Authors:** Giada Crescioli, Mattia Giovannini, Benedetta Pessina, Simona Barni, Antonella Muraro, Alfredo Vannacci, Francesca Mori

**Affiliations:** a Department of Neurosciences, Psychology, Drug Research and Child Health, Section of Pharmacology and Toxicology, University of Florence; b Tuscan Regional Center of Pharmacovigilance; c Allergy Unit, Meyer Children's Hospital IRCCS; d Department of Health Sciences, University of Florence, Florence; e Food Allergy Referral Centre, Padua University Hospital, Padua, Italy

**Keywords:** anaphylaxis, epinephrine, intranasal, pediatrics, pharmacology

## Abstract

**Purpose of review:**

Anaphylaxis is a severe allergic reaction characterized by a rapid onset and can be potentially life-threatening. Epinephrine is considered the first-line treatment, and until recently, it was available only for intramuscular injection. A new intranasal epinephrine delivery device has now been approved for use in adults and children, offering a needle-free option for emergency treatment of allergic reactions. This narrative review explores its technical characteristics, along with its pharmacokinetic, pharmacodynamic, and safety profiles based on the results of the most recent clinical trials.

**Recent findings:**

The key advantages of the intranasal route, including the elimination of needle-length variability, reduced risk of administration errors in obese or underweight patients, and simplified storage requirements, are also discussed. According to recent research, intranasal epinephrine represents an easy-to-use, effective, and well tolerated treatment for severe allergic reactions. Intranasal delivery may offer a painless, easy-to-use, and reliable solution suitable for healthcare professionals, age-appropriate patients and caregivers.

**Summary:**

Based on the current evidence, intranasal epinephrine appears to be a promising, well tolerated option that could significantly improve the accessibility and effectiveness of anaphylaxis management in the pediatric setting.

## INTRODUCTION

Allergic and anaphylactic reactions present major challenges for healthcare providers, patients, and caregivers. Although mild allergic signs and symptoms are treated with corticosteroids and antihistamines, intramuscular epinephrine remains the gold standard for anaphylaxis. In pediatric patients, intramuscular administration can be difficult, especially for self-use, or when administered by caregivers. A new Food and Drug Administration (FDA)-approved intranasal epinephrine device now offers a needle-free option [1]. In this review, we explore the studies that support its approval, data on its efficacy and safety, and its potential role in managing anaphylaxis in pediatrics. 

**Box 1:**
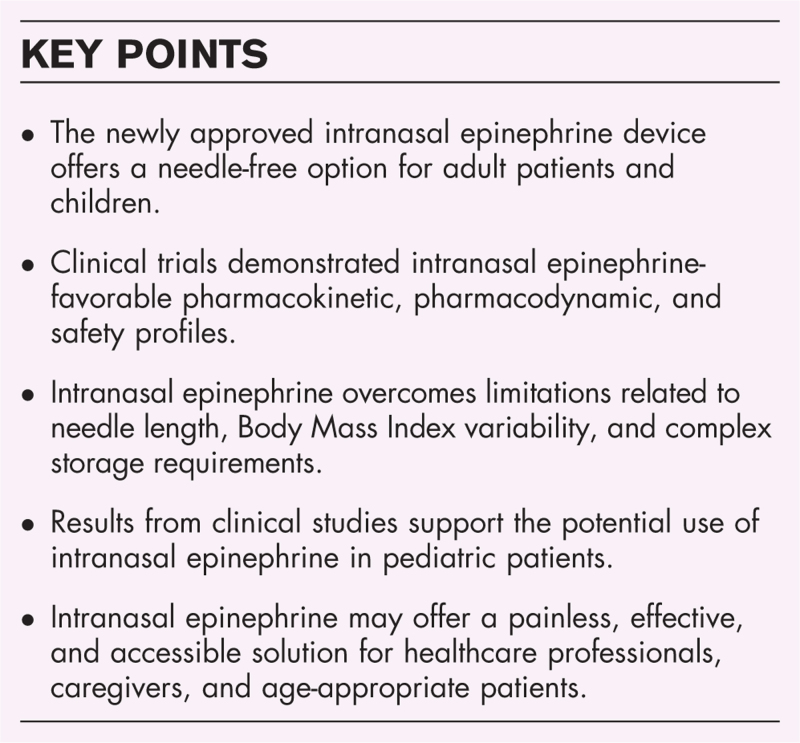
no caption available

## DEFINITION, EPIDEMIOLOGY, AND CLINICAL CHARACTERISTICS OF ANAPHYLAXIS

Anaphylaxis, as defined by the International Classification of Diseases 11th Revision, is a severe systemic hypersensitivity reaction with rapid onset and potentially life-threatening airway, breathing, or circulatory signs and symptoms [2]. Skin or mucosal involvement is common [2–4] but may be absent in up to 20% of cases [5].

Anaphylaxis incidence rates range from 1.5 to 7.9/100 000 person-years, and fatal food-related cases in Europe from 0.03 to 0.3 per million/year [6,7]. The lifetime prevalence is estimated as 0.05–2% in the USA and up to 3% in Europe [7], affecting ~5% of Americans and 0.5% of Europeans [8]. Triggers include food, drugs, and hymenoptera venom [4], with age, asthma, and comorbidities increasing severity risk in adults [6].

Anaphylaxis clinical manifestations vary but usually develop rapidly and affect multiple systems. Common characteristics include urticaria, flushing, angioedema, respiratory distress, hypotension, and loss of consciousness [9,10]. Skin and mucosal involvement occurs in over 90% of cases, while respiratory and cardiovascular manifestations (airway and/or breathing and/or circulation, ABC problems) appear in more than 50% [4,11].

## ANAPHYLAXIS: GUIDELINES FOR CLINICAL MANAGEMENT AND PHARMACOLOGICAL TREATMENTS

In 2021, the Resuscitation Council UK released updated guidelines [3] that emphasize the importance of accurately recognizing anaphylaxis to ensure appropriate pharmacological interventions [11].

Patients must remain flat or semi-recumbent with or without their legs raised to maximize venous return. In fact, standing posture is associated with reduced myocardial filling and perfusion, cardiovascular collapse, and death [12,13^▪^]. Antihistamines are considered a third-line intervention and can be helpful in treating skin signs and symptoms once ABC clinical manifestations are resolved [14]. Additionally, the use of antihistamines is associated with an increased risk of biphasic reactions [15]. Corticosteroids are no longer advised for routine treatment of anaphylaxis, except after initial resuscitation for refractory reactions or ongoing asthma or shock. Prehospital treatment with corticosteroids increases the risk of admission to intensive care by approximately three-fold [16]. These results may be due to the delayed administration of epinephrine, which is considered the first-line treatment for anaphylaxis [11]. Intramuscular epinephrine should be administered during the initial phase of an anaphylactic reaction, with the possibility of an additional dose after 5 min if there is no improvement [3]. Timely administration minimizes the risk of adverse outcomes, including biphasic reactions, hospital admission, and death [17]. Patients who receive prehospital epinephrine have an about 50% reduced risk of having biphasic reactions and shorter emergency department stay [18]. The recommended dose for intramuscular injection is 0.01 mg/kg (maximum 0.5 mg). In adults, this dosage guarantees higher peak plasma concentrations than subcutaneous administration [19]. Only in case of refractory anaphylaxis, continuous epinephrine intravenous infusion with close cardiovascular monitoring may be necessary [8,20].

## ANAPHYLAXIS IN CHILDREN

In children, anaphylaxis is most commonly triggered by food, accounting for up to 66% of pediatric cases, followed by insect venom (19%) [21]. Food triggers vary by age: cow's milk and eggs dominate in infants, tree nuts in preschoolers, and peanuts across all ages. Emergency visits for anaphylaxis have risen sharply, especially in children aged 5–17 [22], and the UK saw a 137% increase in hospital admissions for food-induced anaphylaxis in children under 14 between 1992 and 2012 [23].

Anaphylaxis diagnostic criteria are the same for adults and children, but clinical presentation in pediatric patients also varies by age. Vomiting and cough are more common in the first decade, while subjective clinical manifestations like nausea, throat tightness, and dizziness appear more frequently in older children and adolescents [21]. Cardiac and circulatory manifestations typically present as presyncope and hypotension in adolescents and adults, while in infants and toddlers, they may appear as nonspecific neurological manifestations (e.g. reduced alertness and hypotonia) [24].

For pediatric patients with anaphylaxis, the National Institute of Allergy and Infectious Diseases recommends intramuscular epinephrine as the standard treatment at a dose of 0.01 mg/kg, with a maximum of 0.3 mg for prepubertal children and 0.5 mg for adolescents. If clinical manifestations persist or recur, additional doses may be administered [25]. The Australasian Society of Clinical Immunology and Allergy recommends 0.15 mg epinephrine injectors for children weighing 7.5–10 kg and 0.3 mg for those over 20 kg [26]. Parents and caregivers are strongly encouraged always to carry epinephrine autoinjectors because the event may occur everywhere, including public spaces (e.g. parks and schools) [27–29]. However, parents’ compliance with epinephrine autoinjectors is not optimal. According to a survey, only 29% of participants had an available device, despite 60% reporting that they always carried it; nearly 50% of the devices were expired, and 14% were not correctly dosed. Furthermore, 29% of the patients experienced accidental exposure to food allergens in the previous year, but only 30% of them had epinephrine available at the time of exposure [30,31]. In another study, only 36% of parents in the sample felt confident about using autoinjectors [28]. The main reasons were fear of adverse drug events (ADEs), uncertainty about the severity of the reaction, and difficulties deciding which drugs to use [32]. Moreover, the utility of epinephrine autoinjectors is affected by low prescription fulfilment rates and failure to use due to fear of needles, which is common in children [33]. To address these limitations, research has focused on new epinephrine devices, which may find applicability in frail subgroups, such as children.

## APPROVAL OF INTRANASAL EPINEPHRINE

Sublingual and intranasal administration of epinephrine has been studied since 2015. Preliminary analyses reported comparable pharmacokinetics and pharmacodynamics to intramuscular injection. Regardless of the route, epinephrine is metabolized by catechol-O-methyltransferase and monoamine oxidase, with the metabolites eliminated through the kidneys. In pilot analyses, intranasal epinephrine showed high bioavailability (absorption half-life, 29 min) and reached plasma peak concentration more rapidly than intramuscular injection. Plasma concentrations declined rapidly (elimination half-life, 4.1 min), allowing easy control of exposure in case of adverse events [34]. Significant systemic absorption was observed at a dose of 5 mg, with an average area-under-curve at 0–120 min of 19.4 ng/min/ml and a peak concentration of 386 ± 152 pg/ml [35]. These results represent the starting point for the approval of the FDA's first 2-mg intranasal epinephrine device (date of approval: 09 August 2024) for adults and children weighing at least 30 kg [1]. Recently (05 March 2025), 1 mg intranasal epinephrine was approved for the emergency treatment of type 1 allergic reactions, including anaphylaxis, in children aged 4 years and older, weighing 15–30 kg [36]. Similar delivery systems are already used to administer naloxone, diazepam, nalmefene, and zavegepant [37] to both adults and children.

### Characteristics of the device

The device consists of three components: an active ingredient (epinephrine), an absorption-enhancing agent (dodecyl maltoside, DDM), and a unit dose spray (UDS) [37]. DDM alters the mucosal viscosity and weakens the connections between adjacent cells [38], while the UDS is designed to produce droplets optimized for drug release in nasal turbinates. This combination enables administration of the minimum effective dose of epinephrine, overcoming the possibility of an overdose. Moreover, the low dose administered using the intranasal device reduces the risk of other adverse events, including gastrointestinal ones [39].

### Results of approval studies

#### Pharmacokinetics and pharmacodynamics

In the case of allergic reactions and anaphylaxis, randomized clinical trials (RCTs) encounter several limitations. Allergic reactions cannot be induced; their unpredictable course may place patients in life-threatening conditions [40]. The pharmacokinetics of intranasal epinephrine has been evaluated through blood sample analysis at baseline and multiple time points postdosing. Likewise, pharmacodynamic parameters were recorded at baseline and various intervals postdosing, using an automated device [37].

An integrated analysis of data from four randomized crossover phase 1 trials (175 patients) compared epinephrine pharmacokinetics/pharmacodynamics after the use of manual intramuscular epinephrine 0.3 mg injection, epinephrine 0.3 mg autoinjectors, and epinephrine 1 mg intranasal spray [40]. The maximum plasma concentration achieved with intranasal spray (258 pg/ml) was comparable to that of manual intramuscular injection (254 pg/ml) and lower than that of autoinjectors (Symjepi 438 pg/ml; EpiPen 503 pg/ml). Nevertheless, the intranasal spray demonstrated a more pronounced effect on diastolic blood pressure (DBP), compared with other delivery methods. These findings suggested that intranasal epinephrine administration effectively increases blood pressure at lower plasma concentrations, compared with intramuscular and autoinjector methods.

In a phase 1 randomized crossover study with 59 healthy subjects, 2 mg intranasal epinephrine reached a mean *C*_max_ of 481 pg/ml, intermediate between intramuscular and autoinjector delivery. All treatments increased the systolic blood pressure (SBP) and heart rate (HR), with the strongest effects seen after intranasal administration, even with repeated doses. Intranasal epinephrine levels were associated with SBP and HR for up to 45 and 120 min, respectively. The treatment was well tolerated, with only mild adverse events, supporting its safety and efficacy as a needle-free option [41^▪▪^].

A phase 1 crossover study in adults with allergic rhinitis showed that all participants successfully self-administered intranasal epinephrine, confirming device usability [42]. Intranasal epinephrine reached a higher peak plasma concentration (421 vs. 322 pg/ml) and faster median *T*_max_ (30 vs. 45.0 min) than intramuscular epinephrine. It also produced a greater SBP increase (20 vs. 13 mmHg), with pharmacokinetic/pharmacodynamic responses comparable to or superior to intramuscular administration by healthcare providers. The authors suggested that this may be due to reduced β2-mediated vasodilation.

Oppenheimer *et al.* [43] evaluated whether upper respiratory tract infections (URTIs) impact intranasal administration. The postdose epinephrine concentrations did not differ between healthy patients and those affected by URTIs. Moreover, in patients with URTIs, the analyses highlighted a slight increase in absorption during the first 10–15 min.

Pooled data from four crossover studies highlighted that, after intranasal administration, peak concentrations were reached rapidly (at 25.1 and 20.1 min in the opposite and same nostrils, respectively). Administration in the opposite and same nostrils showed an increase in epinephrine absorption of 55 and 59%, respectively, compared with intramuscular injection. No significant association was observed after intranasal administration according to Body Mass Index (BMI) and sex [44]. Other clinical studies are ongoing [45] or have been completed [46,47], but their results have not yet been published. Table 1 summarizes the key findings of recent trials comparing intranasal and intramuscular epinephrine administration in terms of pharmacokinetic/pharmacodynamic outcomes.

**Table 1:** Summary of key trials comparing intramuscular and intranasal epinephrine treatment

Study	Study type	Population	Treatments	*C*_max_ (pg/ml)	*T*_max_ (min)	Main PD effects
Tanimoto *et al.* [40]	Four phase 1 crossover studies	175 healthy adults	IM manual (0.3 mg), autoinjectors, IN spray (1 mg)	IN: 258 IM: 254 AUT: 438–503	IN: 30 IM: 30 AUT: 20	SBP increase (mean mmHg): IN: 16.9 IM: 10.9 AUT: 18.1 HR (mean beats/min): IN: 13.6 IM: 12.8 AUT: 14.4
Casale *et al.* [41^▪▪^]	Phase 1, 6-treatment crossover study	59 healthy adults	IN (2 mg) vs. IM vs. autoinjector	IN: 481 (mean) IM: 339 (mean) AUT: 753 (mean)	IN: 30 (median) IM: 45 (median) AUT: 7.50 (median)	SBP increase (mean mmHg): IN: 23.6 IM: 11.9 AUT: 18.2 HR (mean beats/min): IN: 17.3 IM: 9.71 AUT: 12.3
Casale *et al.* [42]	Phase 1 crossover study	45 allergic rhinitis patients	IN (2 mg) vs. IM (self-administered 0.3 mg)	IN: 421 IM: 322	IN: 30 IM: 45	SBP increase (mean mmHg): IN, 20 mmHg; IM, 13 mmHg
Oppenheimer *et al.* [43]	Mixed-model analysis study	21 adults with URTI or in normal conditions	IN (2 mg)	URTI: 490 Normal conditions: 570	10–15 min for early absorption	No significant differences between subjects with URTI or in normal conditions
Greenhawt M. *et al.* [44]	Pooled four crossover study	Adults	IN, opposite vs. same nostrils (13.2 mg) IM (0.3 mg)	IN, same nostril: 332 IN, opposite nostril: 262.8 IM: 285.7	IN, same nostril: 20.1 IN, opposite nostril: 25.1 IM: 20	HR (mean beats/min): IN, same nostril: 8.8 IN, opposite nostril: 6.5 IM: 5.9
Fleischer *et al.* [56^▪▪^]	Phase 1, single-dose studies	Children 4–18 years	IN, 1 mg (15–30 kg of weight) IN, 2 mg (>30 kg of weight)	IN, 1 mg: 651 IN, 2 mg: 690	IN, 1 mg: 20 IN, 2 mg: 29.5	SBP increase (mean mmHg): IN, 1 mg: 13.4 IN, 2 mg: 12.2 HR (mean beats/min): IN, 1 mg: 18.5 IN, 2 mg: 16.9

AUT, autoinjectors; SBP, systolic blood pressure; *C*_max_, maximum blood concentration; HR, hearth rate; IM, intramuscular; IN, intranasal; PD, pharmacodynamic; *T*_max_, time to reach the maximum blood concentration; URTI, upper respiratory tract infection.

#### Safety

According to the aforementioned studies, intranasal epinephrine administration was well tolerated, and no significant ADEs were observed. Most ADEs were mild and did not result in withdrawal from the studies [43]. Among reported ADEs, intranasal administration was associated with nasal discomfort and rhinorrhea [42]. Concerns were raised regarding the possibility of intranasal epinephrine-related hypertensive crisis [48]. Intranasal administration is supposed to bypass the interaction with β_2_-adrenergic receptors in the skeletal muscle, while intramuscular administration causes vasodilation of the skeletal muscle vessels, leading to a reduction in venous return and a decrease in DBP without SBP elevation [41^▪▪^]. Epinephrine-related hypertensive crisis is a rare ADE, resulting from the α-adrenergic vasoconstrictive effect of the medication, and can occur in patients of all ages, especially older or suffering from preexisting cardiovascular disease [48]. Due to their nature and peculiar design, RCTs could not detect all ADEs [49]. Thus, further studies will evaluate this issue and clarify the safety profile of intranasal epinephrine administration regarding the potential for hypertensive crisis.

### Advantages of intranasal epinephrine administration: overcoming the issue of needle length, BMI, and difficult storage

Among the advantages of intranasal epinephrine administration, the availability of needle-free devices is certainly of utmost importance. Epinephrine delivery is influenced by multiple interrelated factors, most notably, needle length, sex, propulsion force, and obesity. Needle length plays a critical role in the pharmacokinetics of epinephrine in both intramuscular and subcutaneous injections. Typically, the needle length ranges from 1.17 to 2.5 cm [50] and up to 3.8 cm [11]. Compression applied during injection can also affect the skin-to-muscle distance. In overweight or obese patients and women, an inadequate needle length may lead to subcutaneous rather than intramuscular administration, delaying drug absorption. In fact, female sex and a BMI greater than 30 have been identified as predictors of increased skin-to-muscle depth [51]. Conversely, in children under 15 kg, there is concern about the risk of accidental intraosseous injections [52,53]. These issues are completely avoided with intranasal administration, which utilizes a needle-free delivery system. Another major advantage lies in storage convenience. Unlike autoinjectors, which require controlled storage (20–25 °C) and may lose efficacy with heat exposure [54], intranasal formulations remain stable up to 50 °C [55].

## PERSPECTIVE IN PEDIATRICS

Studies regarding intranasal epinephrine approval have been conducted in adult patients, raising questions about its use in children. A phase I multicenter study enrolled 42 patients aged 4–18 years, who received either 1 or 2 mg of intranasal epinephrine based on their body weight (15–30 and >30 kg, respectively) [56^▪▪^]. The pharmacokinetic/pharmacodynamic data from children were then compared with those obtained from adults. Administration of 1 mg intranasal epinephrine resulted in a slightly lower *C*_max_ and faster *T*_max_, compared with 2 mg intranasal epinephrine, with no significant difference in *C*_max_ between pediatric and adult patients. Moreover, compared with adult patients, in children, the increase of SBP was significantly lower, and DBP showed only minimal differences. Children exposed to 1 mg intranasal epinephrine reported nasal congestion, upper respiratory tract congestion, dry throat, nasal dryness, and paresthesia as the most common adverse events (*N* = 9/42). All adverse events were defined as mild and resolved quickly.

**FIGURE 1:**
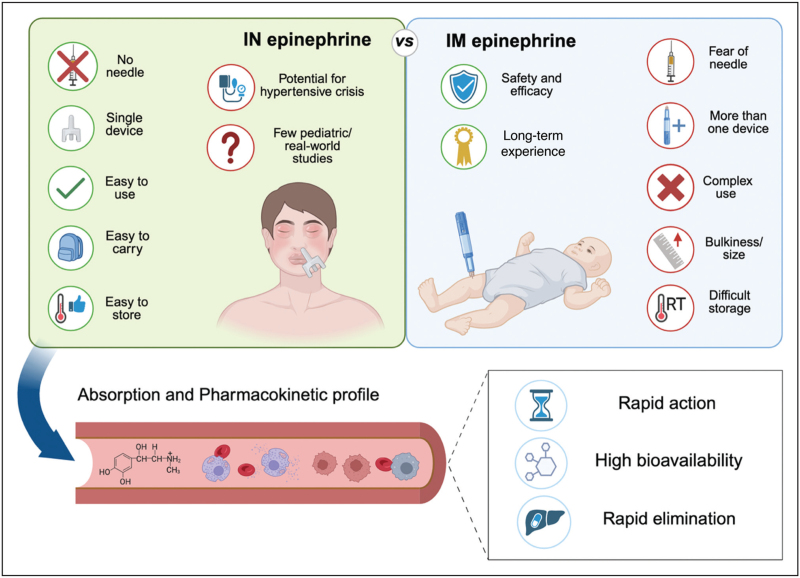
Comparison between intranasal and intramuscular epinephrine. Created with BioRender.com.

The advantages of intranasal epinephrine in pediatrics are already evident (Fig. 1). Intranasal epinephrine sprays are easy to use for healthcare professionals and caregivers, and for age-appropriate patients, needle-free administration may enable self-administration. Moreover, fear of needles and procedural pain, which are particularly significant in pediatrics [57], are entirely avoided with intranasal epinephrine. Regarding pharmacokinetic/pharmacodynamic properties of intranasal epinephrine in children compared with adults, studies are still limited. However, similar to other intranasal drug delivery devices for children (e.g. intranasal fentanyl) [58], intranasal epinephrine has been shown to be effective and well tolerated, with higher compliance and no significant difference in bioavailability [56^▪▪^], despite the fact that adults, compared with children, have a larger surface area for intranasal drug absorption [59]. Therefore, no major differences in the bioavailability are expected in the pediatric population for intranasal epinephrine administration in future studies.

## CONCLUSION

The availability of a needle-free, life-saving epinephrine device marks a major advancement in the management of severe allergic reactions. Intranasal administration may allow timely, painless treatment in children, reduce fear, and improve usability for caregivers, healthcare providers, and age-appropriate patients. Ongoing studies will clarify the benefit–risk and pharmacokinetic/pharmacodynamic profiles in pediatric populations [60]. However, further clinical evidence regarding the comparison between intranasal and intramuscolar epinephrine seems to be required.

## Acknowledgements

*None*.

### Financial support and sponsorship

*None.*

### Conflicts of interest

*M.G. reports personal fees from Sanofi, Thermo Fisher Scientific. S.B. reports personal fees from Nutricia, Sanofi and Firma. The other authors declare that they have no conflict of interests to disclose in relation to this article.*

## REFERENCES AND RECOMMENDED READING

Papers of particular interest, published within the annual period of review, have been highlighted as:

▪ of special interest
▪▪ of outstanding interest
